# Effect of Atropine Eye Drops on Choroidal Thinning Induced by Hyperopic Retinal Defocus

**DOI:** 10.1155/2018/8528315

**Published:** 2018-01-14

**Authors:** Samuel T.-H. Chiang, John R. Phillips

**Affiliations:** ^1^School of Optometry and Vision Science, The University of Auckland, Auckland, New Zealand; ^2^Department of Optometry, Asia University, Taichung, Taiwan; ^3^Department of Medical Research, China Medical University Hospital, Taichung, Taiwan

## Abstract

**Purpose:**

To investigate the effects of atropine on choroidal thinning induced by hyperopic retinal defocus.

**Methods:**

Ten young adults with myopia (−1.00 D to −5.00 D) viewed a video at 6 metres for 60 minutes on successive days. On day 1, one eye (control) was distance corrected with a contact lens; the other (experimental) eye wore a contact lens imposing 2.00 D of hyperopic retinal defocus. Sub- and perifoveal choroidal thickness (SFCT, PFCT) were monitored with optical coherence tomography. On day 2, the procedure was repeated but the experimental eye had received one drop of 0.5% atropine 22 hours earlier.

**Results:**

On day 1, eyes exposed to hyperopic defocus developed progressively thinner choroids (SFCT (baseline) = 253 ± 32 *μ*m versus SFCT (40 mins) = 244 ± 31 *μ*m, *p* = 0.004), whereas SFCT and PFCT in control eyes did not change (*p* > 0.17). On day 2 (22 hours after instilling atropine), baseline SFCT and PFCT were not different to day 1 (*p* > 0.05) and hyperopic defocus failed to thin the choroid (max change in SFCT = +2 ± 2 *μ*m, *p* = 0.36).

**Conclusions:**

Atropine abolished choroidal thinning induced by hyperopic defocus without changing baseline choroidal thickness. The results suggest that atropine inhibits signals associated with hyperopic defocus, for example, from lag of accommodation during near work. This trial is registered with ACTRN12617001519347.

## 1. Introduction

Myopia of any degree increases the risk of developing sight-threatening conditions such as glaucoma, retinal detachment, and myopic maculopathy [1] with risk increasing dramatically with progression to high myopia [2]. Atropine eye drops are currently the most effective treatment for managing myopia progression [3, 4] and they are widely used in Asian countries [5] where the prevalence of myopia is high [6]. Atropine, a nonspecific muscarinic receptor antagonist, is a potent cycloplegic and was initially used for myopia control on the premise that excessive ocular accommodation was the cause of myopia progression. However, subsequent animal research has demonstrated that atropine inhibits myopia progression via a nonaccommodative mechanism. For example, atropine inhibits experimental myopia in the chick without causing cycloplegia [7] and experimental myopia in a mammalian model can be inhibited by the highly selective muscarinic receptor antagonists MT3 and MT7 [8] which do not cause cycloplegia. Moreover, atropine is effective in slowing human myopia progression at very low concentrations (0.01%) that cause negligible cycloplegia [9]. Although atropine inhibits myopia progression by preventing abnormal scleral expansion and eye elongation, its site and mode of action are unclear. Recent evidence from the chick model suggests that the retina is the major site of action of atropine and that atropine likely exerts its action via intermediaries such as nitric oxide (NO) and dopamine [10] although there is also evidence that it may act directly on the choroid [11]. Atropine treatment has been shown to increase the resting thickness of the choroid in animals [11] and humans [12].

The thickness of the choroid is reduced when the retina in animals [13, 14] and humans [15–17] is exposed to hyperopic defocus (image plane located posterior to the retina). Choroidal thickness is also reduced during accommodation [18]. Hyperopic retinal defocus is a consequence of a lag of accommodation which commonly occurs during near work and which has been implicated as one factor in causing eye enlargement and myopia development [19]. The aim of this study was to investigate the effect of atropine eye drops on choroidal thinning induced by hyperopic retinal defocus, using automated measures of choroidal thickness obtained with swept-source optical coherence tomography (SS-OCT). A secondary aim was to record the short-term effect of atropine on the resting thickness of the choroid. Some data reported here has previously appeared in abstract form (Optom Vis Sci 2015;92:E-abstract 150030).

## 2. Materials and Methods

### 2.1. Participants

Ten East Asian adults aged between 18 and 24 (mean ± SD, 20.9 ± 1.8 years) participated in this study, which adhered to the tenets of the Declaration of Helsinki. Ethics approval was obtained from the University of Auckland Human Participants Ethics Committee (Ref 010617), and informed consent was obtained from all participants in writing. The inclusion criteria for this study were as follows: aged 18 to 25, with spherical equivalent refraction (SER) with spectacle prescription between −1.00 and −5.00 D (mean ± SD, −2.63 ± 1.28 D), and with little astigmatism (≤1.00 D) or anisometropia (≤1.00 D). Also, individuals with amblyopia, ocular pathology, or other ocular anomalies (e.g., surgery and trauma) that might have influenced the measurements were excluded. Prior to enrolment, all participants underwent a comprehensive eye examination to confirm their refractive status and to ensure the absence of binocular or pathological abnormalities or history of significant ocular surgery or trauma. All 10 participants had visual acuity of logMAR 0.00 or better.

### 2.2. SS-OCT System and Scan Protocols

The choroidal thickness measurements were obtained using a swept-source optical coherence tomography (SS-OCT) Topcon DRI OCT-1 Atlantis (Topcon Corp., Tokyo, Japan; http://www.topcon.co.jp/), with an axial scan rate of 100,000 Hz operated at the 1 *μ*m wavelength region. The wavelength-sweeping laser had a tuning range of approximately 100 nm centred at 1050 nm, allowing a high axial resolution of 8 *μ*m to be obtained (http://www.topcon.co.jp/). Compared to spectral-domain OCT centred at 800 *μ*m, the longer wavelengths enable deeper penetration of ocular tissues and allow a three-dimensional (3D) high contrast image of the choroid to be obtained. To enhance the signalling and imaging of the choroid, the “chorioretinal” scanning mode of the instrument was selected.

**Figure 1:**
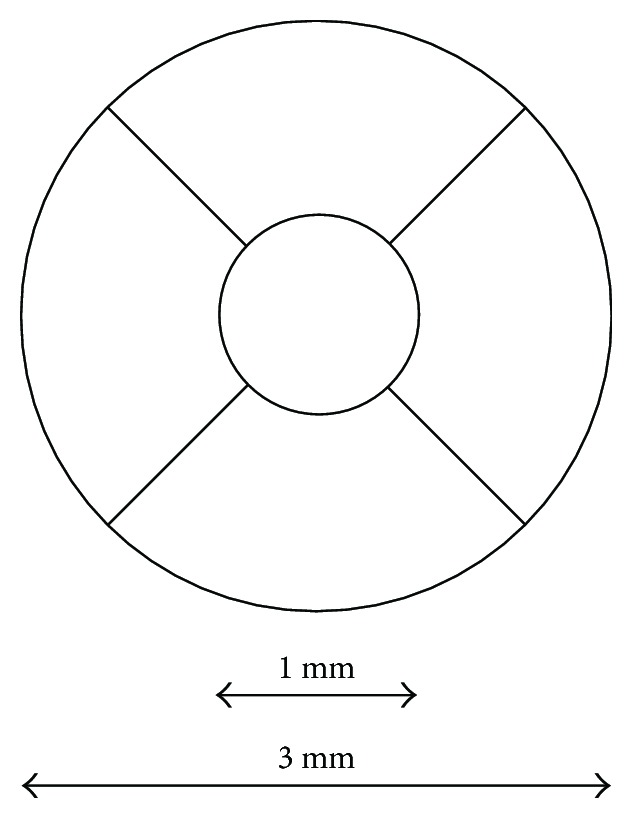
The ETDRS grid overlay centred at the fovea was used to generate automated mean choroidal thickness measures. The central 1 mm diameter circle records the subfoveal choroidal thickness (SFCT). The thickness values within the 4 segments of the 1–3 mm diameter annulus were averaged to record the parafoveal choroidal thickness (PFCT).

A 3D imaging data set covering an area of 6 × 6 mm^2^ centred over the macula was obtained from each participant by using a scan protocol of 512 (horizontal) × 128 (vertical) A-scans per data set. The scanning was carried out by an invisible scanning laser so that eye movements were minimised during the scan. The choroidal thickness was measured by the automatic detection of the outer-border (RPE) and the chorioscleral border in order to avoid experimenter bias influencing the measures. After the choroidal thickness map was obtained from 3D imaging, a grid (Figure 1) used previously in the Early Treatment Diabetic Retinopathy Study (ETDRS) [20] was applied to the map to give automated averaged measures of choroidal thickness within the various segments.

The choroidal thickness within the central 1 mm diameter circle was used as the measure of SFCT. The mean of the values for the 4 segments within the 3 mm diameter annulus were used as the measure of parafoveal choroidal thickness (PFCT).

### 2.3. Experiment Protocol

Participants attended two sessions that allowed choroidal measures to be made at the same time on two consecutive afternoons. At the first visit, 2.00 D of hyperopic retinal defocus was applied to the experimental (nondominant) eye for 60 minutes, while the fellow (control, dominant) eye was fully corrected. Eye dominance was determined using a simple pointing task [21]. OCT measures of choroidal thickness were made in both eyes before applying defocus and at 20-minute intervals during defocus. At the end of the 60 minutes of defocus on day 1, all experimental eyes were treated with one drop of 0.5% atropine. On day 2 (i.e., 22 hours after instillation of atropine), the experimental eye again received 2.00 D of hyperopic retinal defocus for 60 minutes, and the fellow (control) eye was fully corrected. OCT measures of choroidal thickness were made as for day 1.

### 2.4. Stabilisation Period

It has been reported that accommodation [22], exercise [23], and diurnal fluctuations [24, 25] can all cause short-term changes in axial length and choroidal thickness. Therefore, measurements were made at the same time of day for each participant. In addition, prior to each session of measurements and before applying defocus, participants viewed a video movie for 20 minutes (binocular viewing, seated at 6 m from the screen with full distance correction for both eyes) to reduce the influence of previous visual and nonvisual tasks on choroidal thickness.

### 2.5. Monocular Defocus during the Measurement Period

Following the stabilisation period, five consecutive noncycloplegic autorefraction measures were made with an open-field autorefractor (Shin-Nippon NVision-K 5001; http://www.shin-nippon.jp/) to confirm the refractive status of each eye. To induce and maintain the desired level of retinal defocus (2.00 D of monocular defocus), participants wore single vision disposable contact lenses (Johnson & Johnson ACUVUE Oasys; http://www.jnj.com/). For participants who had refractive errors, the desired defocus was combined with the refractive correction into one single contact lens power (e.g., if the distance prescription was −3.00 DS, then to achieve 2.00 D of hyperopic defocus at the fovea in that eye, a −5.00 DS contact lens was given). After contact lenses had settled on the eye (3-4 minutes), five consecutive autorefractor measures were again made in each eye to confirm that the desired refractive status had been achieved, before the measurement period commenced.

Participants then viewed a video movie binocularly at 6 m for 60 minutes while remaining seated and as still as possible. OCT measures were made at 20-minute intervals in both eyes during the 60-minute viewing time. To ensure reasonably large pupil diameters which facilitate rapid OCT measures, the ambient lighting was maintained at about 10 lux (as adopted in previous similar studies [15, 16]) measured with a Digital Light Meter (TES-1335; http://www.tes.com.tw/).

At the end of the first session, trial contact lenses were discarded, and one drop of 0.5% atropine was administered to the lower conjunctival fornix of the participant's experimental (nondominant) eye. The second-day session also consisted of a 20-minute stabilisation period followed by a 60-minute measurement period with the same amount of defocus induced in the experimental eye.

### 2.6. Statistical Analysis

Statistical analyses were performed using IBM® SPSS® Statistics 20. Shapiro-Wilk's *W* test was used to confirm that the data sets were normally distributed. The statistical model employed was repeated measures ANOVA with general linear model (GLM), with 3 within-subject factors: time (0, 20, 40, and 60 minutes), experimental versus control eye, and day 1 versus day 2. Repeated measures ANOVA was run separately for SFCT and PFCT data. Bonferroni-corrected pairwise comparisons were performed for any variables with significant within-subject effect and interactions. A *p* value ≤ 0.05 was considered statistically significant. Values are reported as mean ± 1 SEM (standard error of the mean) unless otherwise specified.

## 3. Results

The mean baseline choroidal thickness within each of the 4 segments comprising the parafoveal annulus (Figure 1) were not significantly different, so they were averaged to give PFCT. The individual baseline SFCT and PFCT varied markedly between participants (e.g., day 1, control eyes: SFCT range: 131 to 483 *μ*m; PFCT range 142 to 444 *μ*m). The baseline SFCT and PFCT in control eyes were very similar for day 1 and day 2 (mean ± SEM: day 1 SFCT = 260 ± 37 *μ*m versus day 2 SFCT = 260 ± 38 *μ*m, *p* = 0.91; day 1 PFCT = 261 ± 33 *μ*m versus day 2 PFCT = 260 ± 33 *μ*m, *p* = 0.44).

On day 1, experimental eyes exposed to 2.00 D hyperopic retinal defocus developed progressively thinner choroids (SFCT at baseline = 253 ± 32 *μ*m versus SFCT at 60 minutes = 245 ± 31 *μ*m; PFCT at baseline = 252 ± 30 *μ*m versus PFCT at 60 minutes = 245 ± 30 *μ*m). The maximum SFCT thinning occurred at 40 minutes when the mean SFCT = 244 ± 31 *μ*m, giving a maximum thinning of 10 ± 2 *μ*m, *p* = 0.004. The maximum thinning of PFCT occurred at 60 minutes when the mean PFCT = 245 ± 30 *μ*m, which equates to a maximum thinning of 7 ± 2 *μ*m, *p* = 0.05. Changes in both SFCT and PFCT in control eyes did not reach statistical significance (SFCT: minimum *p* = 0.17; PFCT: minimum *p* = 0.26).

**Figure 2:**
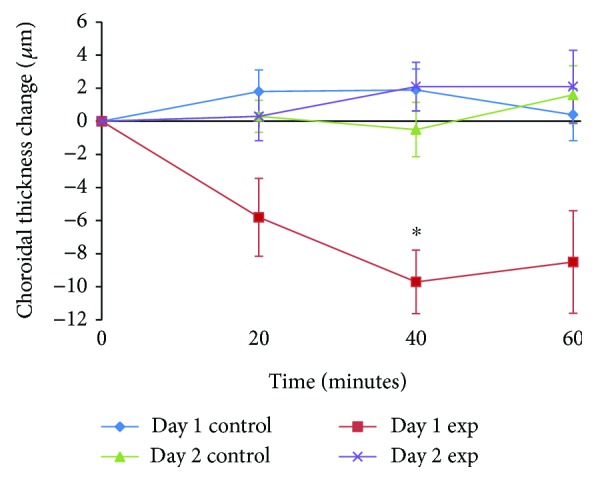
Mean changes in subfoveal choroidal thickness (SFCT) for control and experimental eyes on day 1 and day 2 over the 60-minute testing session. Error bars represent standard error of mean. The asterisk (∗) indicates a significant change in choroidal thickness from baseline (Bonferroni-corrected pairwise comparison *p* ≤ 0.05).

**Figure 3:**
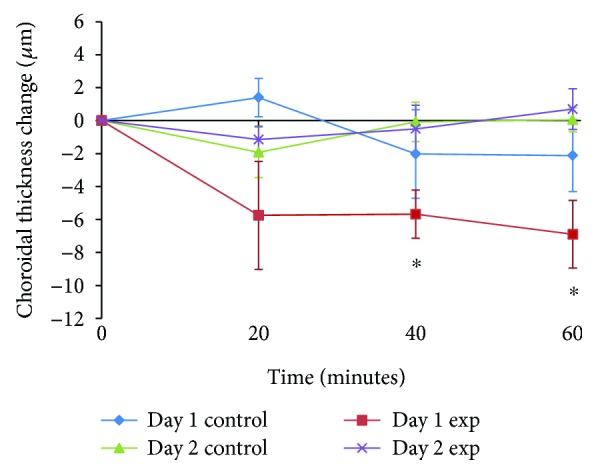
Mean changes in parafoveal choroidal thickness (PFCT) for control and experimental eyes on day 1 and day 2 over the 60-minute testing session. Error bars represent standard error of mean. The asterisks (∗) indicate a significant change in choroidal thickness from baseline (Bonferroni-corrected pairwise comparison *p* ≤ 0.05).

On day 2 (i.e., 22 hours after instilling atropine into the experimental eye), baseline SFCT and PFCT in the experimental eye were similar to those on day 1 (day 1 SFCT = 253 ± 32 *μ*m versus day 2 SFCT = 249 ± 31 *μ*m, *p* = 0.16; day 1 PFCT = 252 ± 30 *μ*m versus day 2 PFCT = 249 ± 30 *μ*m, *p* = 0.09). However, unlike for day 1, 60 minutes exposure to 2.00 D hyperopic defocus failed to thin the choroid (SFCT at baseline = 249 ± 31 *μ*m versus SFCT at 60 minutes = 251 ± 31 *μ*m; PFCT at baseline = 249 ± 30 *μ*m versus PFCT at 60 minutes = 249 ± 30 *μ*m). The maximum change in SFCT occurred at 60 minutes (2 ± 2 *μ*m, *p* = 0.36). The maximum change in PFCT occurred at 20 minutes (2 ± 1 *μ*m, *p* = 0.19).  Figure 2 shows the change in mean SFCT for control and experimental eyes on day 1 and day 2 over the 60-minute testing sessions. The mean changes in PFCT of control and experimental eyes on day 1 and day 2 over the 60-minute testing sessions are shown in Figure 3.

To check for possible regional differences in the effects of defocus and atropine on choroidal thickness within the parafoveal zone, the 4 individual segments were tested separately for the effect of defocus on thickness, the effect of atropine on baseline thickness, and the effect of atropine on the response to defocus. We found no significant differences in these effects between segments.

## 4. Discussion

Previous studies have demonstrated that hyperopic defocus applied to the retina of animals [13, 14] and humans [15–17] causes thinning of the choroid. The primary finding of the current study is that in humans, choroidal thinning in response to hyperopic defocus is abolished by one drop of 0.5% atropine, when measured 22 hours after instillation. This concentration is well within the range of concentrations (0.01–1%) that have been used for myopia control [4].

We also observed a wide range of resting choroidal thickness (prior to atropine) among our participants (range 131 to 483 *μ*m), consistent with other studies [12, 26, 27] that have reported a similarly wide range of choroidal thickness at the fovea (e.g., mean ± SD: 354 ± 111 *μ*m, range 80–641 *μ*m [27]).

Previous studies have also reported that atropine causes an increase in resting choroidal thickness. For example, Nickla and colleagues [11] showed that in chick, intraocular injections of muscarinic antagonists including atropine induced thickening of the choroid 3 hours after injection, even in eyes wearing −10 D lenses. In adult humans, instillation of the homologous drug homatropine 2% also caused an increase in thickness of the choroid within 1 hour of administration [28]. In children, Zhang and colleagues [12] showed that administration of 1% atropine gel twice daily for one week caused an increase in resting choroidal thickness, with the effect being greatest in the inferior meridian. However, in our study, we did not observe an increase (or decrease) in the resting (baseline) thickness of the choroid 22 hours after instillation of 0.5% atropine, even though significant mydriasis was still present and the thinning response to hyperopic defocus was abolished at that time. There are several potential reasons why the choroidal thickening effect of atropine was not observed in our study. It is possible that 1 drop of 0.5% atropine was not potent enough to induce an increase in choroidal thickness. Other possibilities are that either the effect had worn off 22 hours after instillation or less likely that we had not waited long enough to see an effect. A limitation of our study is that we only measured baseline choroidal thickness at one time point (22 hours after instilling atropine) and therefore our data cannot distinguish between these possibilities. Nonetheless, at 22 hours after instillation of atropine, the choroidal thinning response to hyperopic defocus was abolished but the previously reported increase in baseline thickness was not observed. This suggests that these two actions of atropine may have different time courses or can occur independently. Such a conclusion would be consistent with the idea that choroidal thinning and thickening are mediated by different mechanisms as suggested by Nickla et al. [11] on the basis of work conducted in the chick model of myopia. Nickla and colleagues proposed that choroidal thinning is likely mediated via contraction of choroidal, nonvascular smooth muscle by acetylcholine, whereas thickening is via a dopaminergic or nitrergic pathway. On this basis, atropine might block choroidal thinning independently from any direct or indirect effect on a choroidal thickening pathway.

Inevitably, this study has limitations. Apart from the small number of participants (*n* = 10), the effect of atropine was only measured at one time point (22 hours) after instillation. The reason for selecting 22 hours was to ensure that the measures of choroidal thickness were made at the same time of day on the two successive days to avoid any confounding effects of diurnal changes in choroidal thickness [24]. However, the consequence was that we will have missed any shorter-term effects of atropine, should they have occurred, for example, equivalent to the observation that homatropine can cause choroidal thickening 1 hour after instillation [28]. Another limitation of the study is the lack of randomization of the order in which measures with and without atropine were made on the two successive days: measures with atropine were only made on the second day. The reason for instilling atropine only on the second day is that the effects of atropine can be very long lasting (~18 days) [29]. Had atropine been instilled on the first day, then a long washout period (>2 weeks) would have been necessary to ensure that the effects of atropine had worn off by the second measure. Such a long washout period would have been more likely to have allowed other, potentially confounding factors (e.g., changes in environmental temperature and participant health) to affect the results.

## 5. Conclusions

This study shows that one drop of 0.5% atropine abolishes the normal thinning of the human choroid caused by hyperopic retinal defocus, when measured 22 hours after instillation. However, a change in baseline choroidal thickness was not observed with atropine. The inhibitory effect on choroidal thinning suggests that atropine may act to block the myopiagenic effects of hyperopic retinal defocus, for example, with accommodative lag during near work.
